# Genome-scale analysis of Methicillin-resistant *Staphylococcus aureus* USA300 reveals a tradeoff between pathogenesis and drug resistance

**DOI:** 10.1038/s41598-018-20661-1

**Published:** 2018-02-02

**Authors:** Donghui Choe, Richard Szubin, Samira Dahesh, Suhyung Cho, Victor Nizet, Bernhard Palsson, Byung-Kwan Cho

**Affiliations:** 1 Department of Biological Sciences, Korea Advanced Institute of Science and Technology, Daejeon, 34141 Republic of Korea; 2 Department of Bioengineering, University of California San Diego, La Jolla, 92023 CA USA; 3 University of California San Diego School of Medicine, La Jolla, 92023 CA USA; 4 KI for the BioCentury, Korea Advanced Institute of Science and Technology, Daejeon, 34141 Republic of Korea

## Abstract

*Staphylococcus aureus* infection is a rising public health care threat. *S. aureus* is believed to have elaborate regulatory networks that orchestrate its virulence. Despite its importance, the systematic understanding of the transcriptional landscape of *S. aureus* is limited. Here, we describe the primary transcriptome landscape of an epidemic USA300 isolate of community-acquired methicillin-resistant *S. aureus*. We experimentally determined 1,861 transcription start sites with their principal promoter elements, including well-conserved -35 and -10 elements and weakly conserved -16 element and 5′ untranslated regions containing AG-rich Shine-Dalgarno sequence. In addition, we identified 225 genes whose transcription was initiated from multiple transcription start sites, suggesting potential regulatory functions at transcription level. Along with the transcription unit architecture derived by integrating the primary transcriptome analysis with operon prediction, the measurement of differential gene expression revealed the regulatory framework of the virulence regulator Agr, the SarA-family transcriptional regulators, and β-lactam resistance regulators. Interestingly, we observed a complex interplay between virulence regulation, β-lactam resistance, and metabolism, suggesting a possible tradeoff between pathogenesis and drug resistance in the USA300 strain. Our results provide platform resource for the location of transcription initiation and an in-depth understanding of transcriptional regulation of pathogenesis, virulence, and antibiotic resistance in *S. aureus*.

## Introduction

Methicillin-resistant *Staphylococcus aureus* (MRSA) infection is an epidemic health threat and a major worldwide healthcare burden^1–3^. To elucidate the genetic basis of drug resistance, virulence, and pathogenesis, genomes of hundreds of *S. aureus* isolates have been sequenced and analyzed, showing that the characteristics of *S. aureus* are different from strain to strain, even in different strains isolated from the same origin^4–8^. The characteristics of *S. aureus* strains are described in terms of various factors, such as their native host, cytotoxicity, and drug sensitivity^9–11^. Thus, an important question is the genetic bases for community-acquired MRSA (CA-MRSA) to propagate healthy people in a community, become epidemic rapidly, and have different antibiotic susceptibility, compared to healthcare-acquired MRSA (HA-MRSA)^12^. Considering the different history and origin of CA- and HA-MRSA^13^ and the different expression pattern of global regulatory genes between the hypervirulent USA300 lineage and others^14^, the CA-MRSA, especially the USA300 lineage, is expected to have specific transcriptional regulatory networks associated with its high virulence.

Expression and production of virulence factors are tightly regulated by variety of elements such as transcriptional regulators, quorum-sensing, and regulatory RNAs in pathogenic bacteria^15–17^. As in many bacterial species, *Staphylococcal* transcriptional regulators interfere with transcription initiation by binding on *cis*-acting elements located in close proximity of promoter. In addition to transcriptional regulation, 5′ untranslated region (5′UTR) plays pivotal roles in post-transcriptional regulation by containing the Shine-Dalgarno (SD) sequence, that directs ribosome to translate a protein in a specific strength^18^. Regulatory RNA elements, such as riboswitches, on 5′UTR are also important for responding the extracellular and the intracellular stimuli. For example, T-box leader regulates synthesis of aminoacyl-tRNA ligase by sensing cellular level of charged-tRNA^19^. The architecture of transcriptional and translational signals, represented by promoters, *cis*-acting elements, SD sequences, and regulatory 5′UTR sequences, can be identified with various computational toolboxes from experimentally determined transcription start sites (TSSs)^20–22^.

Here, we report transcriptome analysis of methicillin-resistant isolate of community acquired *S. aureus* at Texas Children’s Hospital (TCH) from a pediatric patient (USA300-HOU-MR, also named as USA300_TCH1516; taxid 451516) whose genome sequence was determined in 2007^6^. With the genome-wide determination of TSSs, we identified principal elements of gene expression, such as promoters and SD sequences, regulatory RNA elements, and unannotated transcripts. We also examined strain’s response to antibiotic treatments to investigate genes associated with drug resistance and the regulatory framework that orchestrates gene expression.

## Results

### Determination of polymorphic and hypervariable regions in the genome

**Figure 1:**
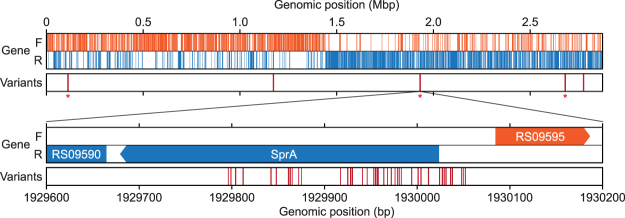
Genomic landscape of sequence variants. Tracks indicate the position of genes in *S. aureus* (gene) and sequence variants (variants). Hypervariable loci (HVL) are marked as (*). 42 variants were mapped on HVL (SprA).

*S. aureus* pulsotype USA300 has been reported to be more virulent than other MRSA strains in epidemiologic studies^1–3^. Also, the strain has subtle genetic changes compared to other CA-MRSA strains^6^. To examine the existence of additional polymorphic variations or mutations, the genome sequence was re-determined (see Methods), which resulted in a total of 68 genomic positions in conflict with the reference genome sequence (NC_010079). The ratio of conflict-containing reads to total reads ranged from 10.0 to 64.3% (“mutant allele frequency” hereafter, Supplementary Dataset S1). The variations were not sequencing error, because error-rate of the sequencing technology is far below the detected frequency^23,24^. Interestingly, we found 66 variants in three genomic regions (97% of the total variants), where we defined as the hyper-variable locus (HVL) (Fig. 1). The first HVL occurred on genomic position from 110,660 to 111,572, containing five variants of two tandem lipoproteins (RS00515 and RS00520), which may be a strain-specific variation^10^. Second, non-coding RNA (ncRNA) *sprA* and 29 nucleotides (nt) upstream region contained 42 variants. Lastly, 19 variants were mapped on the predicted protein (RS13800) and 92 nt downstream intergenic region. Other than HVL, there remained one non-synonymous and one synonymous mutation in the predicted membrane protein (RS05830) and clumping factor B (RS14295), respectively. Particularly, variants on *sprA* and clumping factor B may be an origin of distinct virulence characteristic of the strain, because *sprA* encodes both an ncRNA and a small internal cytolytic peptide^25^, and clumping factor B is related to colonization^26,27^. Further characterization of the two tandem lipoproteins (RS00515 and RS00520) and two predicted proteins (RS13800 and RS05830) is required to widen our understanding of the genomic variations in this strain. This genome information is also essential to identify the transcription regulatory elements for understanding the effects of genomic variations on the bacterial phenotype.

Analysis of short-read sequencing reads on the reference genome is limited in its ability to detect the acquisition of external genetic materials or lateral/horizontal gene transfers, because sequencing reads of the acquired genes are simply discarded due to the reference-guided read mapping. To test this, we examined the acquisition of genetic materials using *de novo* assembly of 6,210,262 sequencing reads (total 29 assembled contigs). All of the contigs were mapped on the reference genome without gap or macro-scale genome rearrangements, indicating no external genetic materials were acquired (Supplementary Fig. S1).

### Landscape of the primary transcriptome

**Figure 2:**
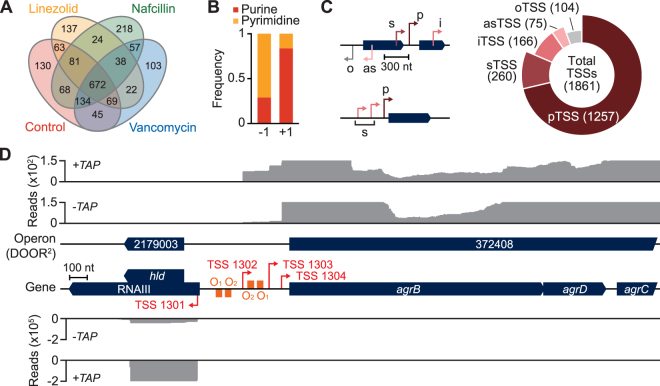
Primary transcriptome architecture of *S. aureus* USA300 TCH1516. (**A**) A total of 1,861 TSSs determined from four different experimental conditions were compared in condition-specific manner. (**B**) TSS (+1) was occupied with purine bases, while pyrimidine was enriched at −1 position. (**C**) Categorization of TSSs according to their relative position and direction to the downstream gene. TSSs positioned upstream (within 300 nt) of a gene with the same direction were annotated as primary (pTSS) or secondary (sTSS). TSSs located inside a gene without downstream gene in close vicinity (<300 nt) were annotated as internal (i) with the same direction of containing genes, or antisense (as) with the opposite direction against the containing genes. TSSs located intergenic region without cognate gene were annotated as orphan (o). (**D**) AgrBDCA operon was transcribed by a primary TSS and two secondary TSSs. Two Agr operator sites are located upstream of the primary TSS.

The primary transcriptome analysis provides information on the structure of transcription unit (TU) as well as a wide array of regulatory elements encoded in the genome, including alternative TSSs, promoters, and 5′UTRs^28,29^. The following three antibiotics, which the strain is sensitive to, were selected to reflect environmental perturbations of special interest: linezolid (**L**), oxazolidinone that inhibits translation initiation complex formation; nafcillin (**N**) and vancomycin (**V**), β-lactam antibiotic and glycopeptide, respectively that inhibit cell wall synthesis. We conducted the minimum inhibitory concentration (MIC) assay to determine the sub-inhibitory concentration of each drug, i.e. ¼ MIC, to use in the antibiotic stress studies. The MIC values determined were as follows: nafcillin = 10 μg ml^−1^ (resistant defined as >4 μg ml^−1^ per the Clinical and Laboratory Standards Institute, CLSI); vancomycin = 2.0 μg ml^−1^ (susceptible defined as <2 μg ml^−1^ by CSLI); and linezolid = 1.0 μg ml^−1^ (susceptible defined as <4 μg ml^−1^ by CSLI). (Supplementary Fig. S2). Using the rRNA-depleted total RNAs prepared from the antibiotics treated samples, we experimentally determined TSSs using the differential RNA-seq (dRNA-seq) method^30^. 5′ ends of primary transcripts were selectively determined (see Methods), identifying a total of 1,861 TSSs from the different antibiotics treatment conditions (Fig. 2A and Supplementary Dataset S2). Similar to the dinucleotide sequence conservation of a wide variety of bacteria^29,31–34^, 83.8% of 1,861 TSSs were occupied by purine bases and pyrimidine base was strongly preferred at −1 position (71.1%) (Fig. 2B). The determined TSSs showed strong agreement with the sequence properties of previously identified sites of transcription initiation (Supplementary Fig. S3). Independent verification of the TSS mapping for five genes was also obtained by a conventional 5′-rapid amplification of cDNA ends (5′RACE) method (Supplementary Fig. S4). Based upon the current genome annotation (2,838 annotated genes in total), each TSS covers 1.52 genes.

The TSSs identified were further classified into five different types according to their positions and direction relative to the cognate genes as described in the previous reports (Fig. 2C)^29,33,35^. First, 1,257 primary TSSs (pTSS) were assigned, when the TSS is located within 300 nt upstream of the currently annotated genes, which corresponds to 44.1% of the total genes in the *S. aureus* genome (note that the monocistronic and operonic structure have not been considered). By integrating the detected TSSs with 1,636 computationally predicted operons in the DOOR^2^ database^36^, we identified primary TSSs for 1,022 operons (62.5%). Among them, 521 were multi-gene operons consisting of 1,578 genes and other operons are transcribed from respective single genes. In addition, 260 secondary TSSs (sTSS) as alternative TSSs to primary TSS were identified from upstream regions of 225 genes, revealing that transcription of 235 operons was initiated by more than one TSS. A total of 166 TSSs were mapped within 136 genes (internal TSS, iTSS) and 104 TSSs were also detected in the intergenic regions with no associated genes (orphan TSS, oTSS). 75 TSSs mapped in the antisense strand of 64 genes (antisense TSS, asTSS), suggesting that presence of regulatory antisense RNAs^25,37,38^ (Supplementary Fig. S5). Compared to the previously detected TSSs of *S. aureus* MW2^31^, 1,235 TSSs were identical and 626 TSSs were newly detected in our study (Supplementary Dataset S2).

Particularly, the virulence and antibiotics resistance genes constituted 134 operons that were frequently located in the mobile genetic elements and pathogenicity islands. Among those, we found the primary TSSs for 63 operons and the additional 13 alternative TSSs for 11 operons, which comprised of total 76 TUs. Those include accessory gene regulator *agrBDCA*, virulence factor *ear*, and plasmidal β-lactamase *blaRI* (Supplementary Dataset S3). For instance, in Agr locus, where δ-hemolysin, RNAIII, and *agrDCBA* operon are encoded, a primary TSS of *agrDCBA* operon (TSS_1304) was observed as in the previous study (Fig. 2D)^39^. Interestingly, two additional secondary TSSs were identified from the upstream of the operators (TSS_1302) and at the overlapping region with the two operators (TSS_1303), respectively. Transcription of *agrDCBA* operon is positively regulated by AgrA-binding *cis*-acting operators, AgrA O_1_ and AgrA O_2_, which are located at the upstream of the TSS_1304^40^. These two additional TSSs are probably responsible for basal expression of *agrDCBA* for priming quorum-sensing system. Thus, the alternative TSSs potentially play pivotal roles in transcriptional regulation^41^ of the virulence and pathogenesis-related genes.

### Elucidation of novel transcripts based on the analysis of 5′ upstream sequences

**Figure 3:**
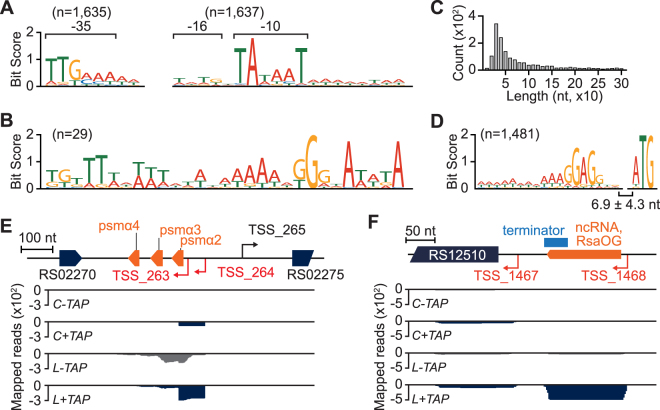
Categorization, alternative transcription, and analysis of 5′-UTRs in *S. aureus* USA300 TCH1516. (**A**) Conserved prokaryotic -35, -16, and -10 promoter elements were found in 1,638, 1640, and 1640 TSSs with *p*-value less than 0.05 (MEME), respectively. (**B**) A conserved SigB binding sites were found from 29 TSSs (*p*-value of 1.51 × 10^−8^). (**C**) Distribution of 5′UTR length of primary and secondary transcripts. Over 75% of TSSs have 5′UTR length below 100 nt. (**D**) AG-rich Shine-Dalgarno sequence (SD) was found from 5′UTR of 1,481 protein-coding transcripts. The spacer length between start codon and SD sequence showed mean length of 6.9 nt. (**E**) An orphan TSS (TSS_263) transcribes novel transcript encoding PSMα2, 3, and 4. (**F**) A transcriptional terminator was predicted at downstream of the TSS_1468. The short transcript generated by TSS_1468 encodes a novel ncRNA RsaOG conserved in *S. aureus*.

The conserved promoter elements such as -10 and -35 sequences are critical components for the determination of transcription efficiency of individual genes. To determine the promoter elements, we analyzed the sequences from 60 nt upstream to 10 nt downstream of each TSS. The conserved -10 (TATAAT) and -35 (TTGAAA) elements were found in 87.9% (1,637 out of 1,861; *p* < 0.05; MEME) and 87.8% (1,635 out of 1,861; *p* < 0.05; MEME) of the identified TSSs, respectively (Fig. 3A and Supplementary Dataset S4). Genes regulated by those promoters are expected to be a regulon of the housekeeping sigma factor (SigA) and the promoter motif is quite similar with that identified in the previous studies^31,42^. The most frequent distance from -10 element to TSS was 7 nt in both *S. aureus* USA300 and *S. aureus* MW2^31^. Total 204 promoters contained the extended -10 element (TRTG), which is less conserved than that in *S. aureus* MW2. Nevertheless, similar to other bacterial promoters, the variable length of spacer sequence between -10 and -35 elements (mostly, 17 ± 1 nt) probably reflects the sigma factor diversity in the *S. aureus* USA300 genome^43–46^.

Additionally, we determined another motif from the 5′ upstream regions of 26 TSSs with *p*-value less than 1.66 × 10^−5^ (Fig. 3B). Along with the high sequence homology between *Staphylococcal* and *Bacillus* SigB^47^, high similarity of the motif with SigB promoters in *B. subtilis* (*p*-value of 1.51 × 10^−8^, MEME) (Prodoric release 8.9; accession MX000073)^48^ shows the potential recognition of those promoters by SigB (RS11145) (Supplementary Fig. S6). The SigB promoter motif was very similar with previously determined SigB promoters^31,42^. Moreover, 19 out of 26 promoters regulate genes related to stress response and virulence such as possible stress protein (RS09240) and staphylocoagulase (RS01175), consistent with the known regulon of *Staphylococcal* SigB (Supplementary Dataset S4)^42,49–51^. In addition to the promoter structure, that affects transcription, 5′UTR of bacterial mRNA is important to control translation efficiency of a gene^52^. 5′UTR length of the protein-coding transcripts had median length of 44 nt and the frequent size range of 30–39 nt, similar to the other bacterial 5′UTR lengths (Fig. 3C)^34,53^. From most of transcripts (n = 1,481), we found a conserved AG-rich SD sequence (AAAGGAGG) in the upstream region of start codon with a mean spacer length of 6.9 nt (Fig. 3D).

The presence of regulatory sequences and mis-annotation tend to increase the length of 5′UTR, with the result that some TSSs had 5′UTR length longer than 300 nt cutoff and could not be assigned to a gene. Thus, we examined the downstream sequence of iTSS, asTSS, and oTSS using Rfam search^22^. In parallel, the sequences were also subjected to BLAST search to find the unannotated or mis-annotated proteins in the long 5′UTR. We found a total of 15 novel transcripts including phenol soluble modulin α (PSMα; Fig. 3E), non-coding RNA (ncRNA), *cis*-regulatory elements such as T-box leaders, transcriptional attenuators, riboswitches, signal recognition particle (SRP), and ribozymes (Supplementary Dataset S5). The three PSMα, which were recently highlighted as responsible for higher cytolytic activity in CA-MRSA^54^, but ignored by computational annotation due to their extremely small size^55^, were highly expressed in linezolid condition (Supplementary Fig. S7**)**.

Due to the extremely small size (less than 70 bp), the computational gene annotation failed to annotate the PSMα^55^. In addition, there is a possibility that unannotated gene between sTSS and pTSS causes mis-assignment of pTSS to sTSS. To elucidate this possibility, we examined the presence of terminator sequences on 5′UTR of 260 sTSS using ARNold software (Supplementary Dataset S5)^56,57^. For example, a rho-independent terminator was located between the primary (TSS_1467) and the secondary TSS (TSS_1468) (Fig. 3F). Thus, the TSS_1468 is a primary TSS where the transcription of Staphylococcal ncRNA RsaOG (Rfam accession RF01775) is initiated^58^. Taken together, the determination of TSSs elucidates the regulatory elements encoded in the promoters and 5′UTRs as well as the presence of novel transcripts, which are potentially relevant to the virulence and antibiotics resistance of the strain.

### Changes in gene expression levels in response to antibiotic treatment

With the experimentally curated TU annotation and newly annotated genes, we profiled the transcriptome changes of the USA300 strain in response to exposure to the three chosen antibiotics (**L**, **N**, and **V**). Strand-specific RNA sequencing (ssRNA-seq) was performed on the rRNA-depleted total RNAs prepared from the antibiotics treated samples (Supplementary Dataset S6). After normalization of the unique mapped reads for each gene, we verified a high degree of correlation between biological replicates by pairwise correlation coefficient (R^2^ > 0.86) and principal component analysis (Supplementary Fig. S8). Furthermore, independent quantitative PCR analysis was conducted to demonstrate the validity and reproducibility of the ssRNA-seq experiments (Supplementary Fig. S8).

**Figure 4:**
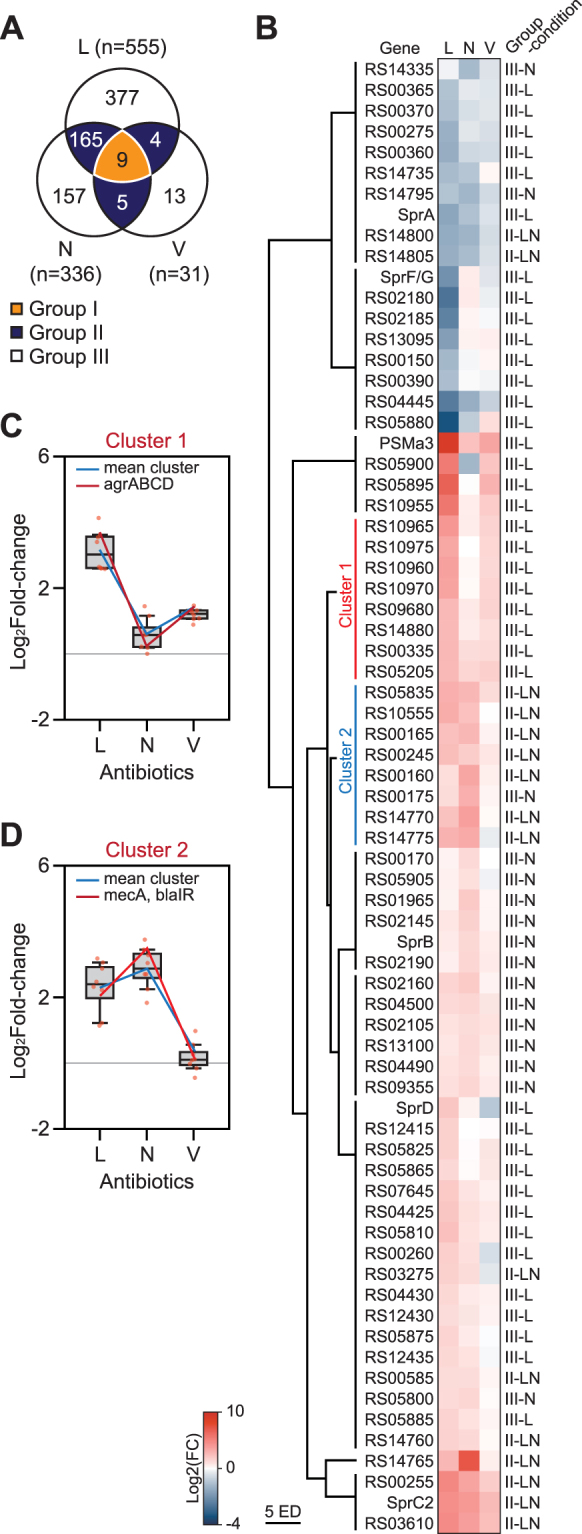
Three different groups of differentially expressed genes (DEGs) and hierarchical clustering of differentially expressed virulence and antibiotic-resistance genes. (**A**) Three large gene groups were determined from 730 DEGs according to their expression in response to antibiotic treatments. Group I, II, and III include 9, 174, and 547 genes, respectively. Group I genes responded to all three antibiotics treated. Group II genes responded two of the three treatments. Group III genes responded uniquely to treatment. (**B**) 71 genes related to antibiotics resistance or virulence were differentially expressed. They were further clustered into 10 clusters according to their expression patterns. Heatmap indicates Log_2_ fold-change of expression levels compared to control condition. (**C**) 8 genes include *agrBDCA* in cluster 1. (**D**) Another 8 genes include *mecA*, *blaI*, and *blaR* in cluster 2. Two clusters were negatively responded against antibiotics (L: Linezolid/control, N: nafcillin/control, V: vancomycin/control).

We then obtained a list of differentially expressed genes (DEGs) between the conditions that satisfied the criteria of at least a two-fold or greater change in gene expression and a *p*-value cutoff (DESeq2 *P* < 0.01). Considering the operon where the DEGs are located, the **L**, **N**, and **V** treatments resulted in 555, 336, and 31 DEGs in 396, 236, and 27 operons, respectively (Supplementary Dataset S7). The DEGs (730 genes in total) formed three large groups in which 9, 174, and 547 genes were differentially expressed in response to all three antibiotics (universal response; group I), two antibiotics (bi-specific responses; group II), and a single antibiotic (unique responses; group III), respectively (Fig. 4A and Supplementary Fig. S9).

The genes in the group I encode the competence protein ComF, predicted acetyltransferase, and predicted phospholipase. The ComF is a competence protein that uptakes exogenous DNA probably playing an important role in the horizontal gene transfer^59,60^. Interestingly, the *comF* was positively regulated over 6.3-fold in all three conditions, demonstrating upon antibiotics treatment the potential strategy of strain to develop a multi-drug resistance. Group II consists of 165, 4, and 5 genes differentially expressed under the **L**-**N**, **L**-**V**, and **N**-**V** treatments, respectively; whose functions are diverse. The transcription of 377, 157, and 13 genes in the group III showed antibiotic-specific induction or repression under **L**, **N**, and **V** treatment, respectively, such as *agrBDCA* gene for regulating virulence in response to **L** treatment. Although the expression patterns clearly showed that many genes in the group III are uniquely required for the response to each antibiotic, the functions of genes in the group I and II are could be involved in the general response to antibiotics treatments.

Next, we examined the expression of virulence and antibiotics resistance genes frequently located in mobile genetic elements (MGEs) and pathogenic islands. Among the total 233 genes in the 134 operons (Supplementary Fig. S9), we determined 71 DEGs across the experimental conditions (Fig. 4B). Most of the virulence genes were regulated in condition specific manner (n = 55) and there was no universal response to all three antibiotic treatments detected. For example, expression of ncRNA SprA was specifically inhibited by **L**. Although the genes have been categorized into three groups (i.e., universal, bi-specific, and unique), individual genes in the same group showed different expression levels. For example, the expression levels of genes encoding exfoliative toxin A (RS05880) and phenol soluble modulin β1 (RS05895) in the group III were decreased by 11-fold and increased by 107-fold uniquely in response to **L** treatment (Fig. 4B). Thus, we further subdivided 71 virulence genes into 10 clusters using hierarchical clustering (Supplementary Dataset S8 and Supplementary Fig. S10). Interestingly, we discovered two clusters, which their expression levels seemed to be reciprocal in response to the antibiotics treatments (Fig. 4B). Cluster 1 consists of 8 virulence genes including full operon of accessory gene regulator (Agr) (Fig. 4C). The Agr and Sae systems control the majority of secreted proteins, including toxins, during pathogenesis^61,62^. As a consequence of Agr induction by **L** treatment, its secreted toxin regulons, such as hemolysins^62^, leukocidins^63^, and PSMs^62^ (Supplementary Fig. S7), are highly induced upon the **L** treatment (Fig. 4B and Supplementary Dataset S6). Meanwhile, expression of the Agr system and exotoxins remained low by **N** and **V** treatments. Interestingly, cluster 2 contained penicillin-binding protein MecA with additional seven virulence genes (Fig. 4D). We observed that MecA and Agr operon have negative correlation in their expression levels. Although the detailed mechanism is still unknown, it has been hypothesized that Agr system and methicillin resistance are inter-connected^64,65^. We identified the DNA binding motif of MecA-regulator MecI and BlaI in Agr locus. The motif located on AgrA binding motif in Agr locus with 65% of similarity (Supplementary Fig. S11), indicating crosstalk between virulence and antibiotics resistance regulation in the strain.

### Metabolic regulation of virulence regulator revealed by pathway enrichment analysis

**Figure 5:**
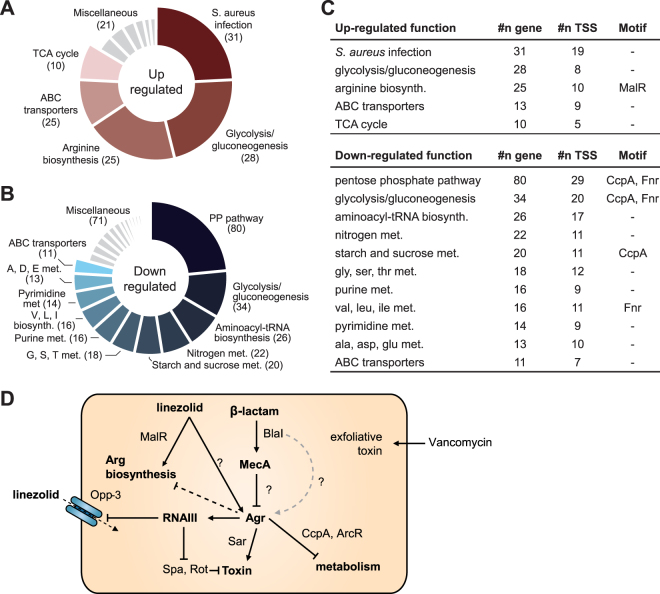
Structure of Agr locus and proposed regulatory network established by the Agr and Mec/Bla systems. (**A**) According to pathway enrichment analysis, genes composing glycolysis, arginine biosynthesis, and TCA cycle were enriched in up-regulated DEGs. (**B**) Meanwhile, pentose phosphate (PP) pathway, amino acid and nitrogen metabolisms were enriched in down-regulated DEGs. (**C**) MalR, CcpA, and Fnr binding motifs were predicted from the pathways. (**D**) Proposed regulatory network by Agr and Mec/Bla system. Molecular mechanism of Agr induction by linezolid is unknown. MecA is proposed to inhibit the Agr system by potential binding on Agr locus. AgrA induces its downstream virulence determinants, while inhibiting expression of metabolic enzymes possibly with CcpA and ArcR. Linezolid possibly induces the expression of arginine biosynthesis pathway by MalR. This induction seems to override Agr mediated repression of arginine biosynthesis^62^.

The isogenic *agr* mutant demonstrated interconnection between Agr signaling and metabolic enzymes^62^. Therefore, cellular metabolism was expected to be changed according to upshift of Agr expression by linezolid. In this context, we analyzed the changes in gene expression related with metabolism in response to linezolid treatment by mapping DEGs on KEGG pathway (Fig. 5A–C and Supplementary Fig. S12). The genes related to carbon metabolism, such as pentose phosphate pathway, glycolysis/gluconeogenesis, starch and sucrose metabolism, purine and pyrimidine metabolism, and metabolism of various amino acids, were down regulated. We observed two *cis*-acting regulatory motifs on the promoter regions of down regulated genes, possibly providing a molecular link between metabolism and Agr system (Fig. 5C and Supplementary Fig. S13). First, CcpA box, a DNA-binding site of conserved gram-positive catabolic control protein A (CcpA) was found at 22 TSSs of pentose phosphate (PP) pathway, glycolysis/gluconeogenesis, and sucrose metabolism with a *p*-value less than 2.64 × 10^−5^ (MEME; Supplementary Fig. S13A). The strain possesses CcpA (RS09215), whose expression was upregulated by 1.71-fold (*p*-value = 7.75 × 10^−4^, DESeq2) on linezolid treatment. Second, Fnr binding motifs were found with *p*-value < 1.63 × 10^−6^ (MEME) at 10 TSSs transcribing pentose phosphate pathway, glycolysis/gluconeogenesis, and branched-chain amino acid biosynthesis genes (Supplementary Fig. S13B). The strain has Crp/Fnr-like transcriptional regulator ArcR (RS14300) without significant change in expression (*p*-value = 0.515, DESeq2). The DNA binding site of ArcR was identified in *S. aureus* SH1000, which is the same as the Fnr motif^66^. To sum up, considering the linezolid-derived perturbation of *S. aureus* metabolism, although it is unclear that the perturbation was induced directly by Agr system, CcpA and ArcR may regulate metabolic genes cooperatively with or at the downstream of Agr regulatory system.

In contrast to down-regulated metabolic genes described above, central carbon metabolism (glycolysis/gluconeogenesis and TCA cycle) and arginine biosynthesis were up-regulated by linezolid. In the previous report, Agr system down-regulates metabolic genes related to carbohydrate and amino acid metabolism (including arginine biosynthesis)^62^. Arginine is an important metabolite in the host defense mechanism by generating nitric oxide (NO) and ornithine. NO has antimicrobial properties and polyamines synthesized from ornithine play critical roles in tissue repair and anti-inflammatory responses in the host^67^. During pathogenesis, the host establishes a low-arginine condition by immune response. Thus, we speculate that up-regulation of arginine biosynthesis may counteract arginine limitation established by the host cell and override regulation by Agr in USA300. This regulatory response could be mediated by MalR-like transcription factor as the MalR-binding motif was found at the upstream regions of arginine biosynthetic genes (*p*-value < 1.41 × 10^−7^, MEME) (Supplementary Fig. S13C).

## Discussion

We experimentally determined transcriptomic responses of the *S. aureus* USA300-HOU-MR against multiple antibiotic treatments. This data yields three important overall results.

First, the primary transcriptome architecture of the strain was determined based on a total of 1,861 TSSs detected under the four experimental conditions used. Over two hundred of genes were transcribed by multiple TSSs, suggesting that *S. aureus* USA300 has a complex regulatory network at transcription level. The strain had promoters composed of -35, -16, and -10 bacterial promoter elements (TTKMAA, TNTK, and TAWAAT) recognized by a housekeeping sigma factor SigA. Spacer sequences between -35 and -10 elements were highly variable in the strain. Four alternative sigma factors have been intensively studied in *S. aureus*^31,43,44,68^. Among them, SigB and SigH have a distinct promoter sequence specificity compared to the SigA^31,44^. However, it is unclear how SigA and exocytoplasmic sigma factor SigS discriminate the promoters, because promoter elements recognized by those two alternative sigma factors are similar with those of SigA^43,68^. In *E. coli*, a spacer between the -35 and -10 region has an important role in promoter recognition by SigD and SigS to transcribe the genes in their regulons^69^. Thus, the variable length and sequence of the spacer may have similar function in *S. aureus*. The regulatory RNA elements such as riboswitches and ribozymes were detected from 5′UTR regions of orphan TSSs.

The primary transcriptome can be used to find novel transcripts including protein-coding genes or ncRNAs^70,71^. For example, novel transcripts encoding PSMα and ncRNAs were also detected in our study, which have not been annotated previously. Surprisingly, the expression of PSMα3 was increased over 526-fold when exposed to linezolid with false-discovery rate of 1.16 × 10^−16^, indicating that the important role of PSMα3 in the pathogenic response to the linezolid like those of other toxins in the strain. Also, secondary TSSs were often assigned to primary TSSs for the precedent ncRNAs, of which one novel ncRNA showed high similarity with ncRNA RsaOG that has been reported to have regulatory function to toxins. The other transcript showed no similarity with any known functional RNAs, while it is highly conserved in more than 100 *S. aureus* strains. Further identification will widen our understanding of virulence regulation by variety of functional RNAs in *S. aureus*. Transcription initiation by secondary TSSs may bypass transcriptional regulation by escaping from *cis*-regulatory elements. In our calculations, there was no difference between primary and secondary TSSs in terms of purine occupancy at +1 site and structure-forming free energy of 5′UTR^72,73^. Overall, the secondary TSS may play an important role in transcriptional regulation with sequence and location specific manner.

Second, RNA-seq reveals plasticity of gene expression changes in response to different antibiotics. Interestingly, the USA300 strain had universal response characteristics under the three different antibiotic treatments. For example, the gene encoding the competent protein (ComF), which is responsible for DNA uptake, was induced when exposed to antibiotics. The induction may play a key role to develop multi-drug resistance by horizontal gene transfer upon contact with antibiotics. The strain also exhibited unique responses to different antibiotics. Upon linezolid treatment, expression of various genes related to virulence, such as enterotoxins, hemolysins, PSMs, and Agr system, were increased. However, expression levels of fibrinogen-binding protein, α-hemolysin, macrolide and aminoglycoside resistance genes were maintained regardless of antibiotics. Interestingly, oligopeptide transporter Opp3 was down regulated by linezolid. The transporter is related to antimicrobial peptide sensitivity of the strain and induces dramatic increase in antimicrobial peptide GE81112 sensitivity^74^. If linezolid is transported into a cell via the Opp system by its peptide-bond moiety and repression of Opp3 system was due to linezolid response of the strain, Opp3 and its regulation system can be a potential drug target. Opp3 agonist and drugs that induce expression of Opp3 could be potential drugs as an adjuvant, increasing effect of the linezolid. A set of enterotoxins in νSAα, serine protease and lantibiotic-synthetic genes in νSAβ, and superantigen-like proteins in νSAγ, were not expressed in any of the experimental conditions^11,16,75^.

Third, systematic analysis including pathway enrichment and expression pattern clustering elucidated the regulatory and metabolic frameworks of the strain. Many virulence factors and metabolic enzymes are regulated by Agr system. Upon the linezolid treatment, Agr system was highly induced and its downstream regulons were up regulated. Meanwhile, variety of metabolic pathways, such as PP pathway, amino acid metabolism, purine and pyrimidine metabolism, and nitrogen metabolism, were down regulated. In addition, Agr system was repressed by nafcillin treatment, possibly by interference of β-lactam resistance. Agr and Bla/Mec systems in USA300-HOU-MR lineage have trade-off relationship to exploit cellular resources for pathogenesis or antibiotic resistance. By inventing the inducible β-lactam resistance that shuts down production of virulent factors, the strain is able to reallocate its proteome to maximum resistance (Fig. 5D). We proposed the signaling hierarchy of gene regulators based upon the previous reports^76^ and potential MecI-binding site at the upstream of *agr* locus. Elucidation of *cis*-regulatory elements of the strain suggests the downstream actuator of gene regulation, such as CcpA and ArcR, governed by Mec/Bla and global regulator, Agr. We also showed transcription factor binding motifs enabling the alternative transcription in Agr locus, which may explain the interplay between the transcription factors. Considering antagonistic relationship between Agr and Mec/Bla systems, it is questioned that how expression of Agr system will be changed by treating linezolid and nafcillin at the same time. Thus, combinatorial treatment of both linezolid and nafcillin is a promising method to understand epistatic interaction between transcriptional regulators in the strain.

We provide a comprehensive repertoire of experimentally verified primary transcriptome information with their regulatory elements in *S. aureus* USA300. Comparative analysis of differential transcriptome analysis revealed transcription architecture and regulatory framework of MRSA. Importantly, taken together, our results suggest the interconnected and tradeoff relationship between antibiotic resistance determinant, virulence regulators, and metabolism, and thus the need for systems analysis of pathogen physiology and responses to therapeutic interventions.

## Methods

### Bacterial strain and culture

*Staphylococcus aureus* strain aureus substr. USA300-HOU-MR (also named USA300_TCH1516, taxid: 451516) was used in this study. Cells were grown on Todd Hewitt Broth (THB, Hardy Diagnostics). Cells were primed overnight with sub-inhibitory amount of nafcillin (2 μg/mL of final concentration), prior to all experiments. For antibiotic sensitivity assay, overnight grown cell culture was diluted 1/40 fold into fresh THB and grown to logarithmic growth phase (OD_600 nm_ of 0.4). The bacterial culture was washed in THB and added to individual wells of a 96-well plate containing 195 μL of media and serially diluted antibiotics. The initial concentration of bacteria was 1 × 10^5^ CFU/well. The plate was incubated for 24 h at 37 °C, and the absorbance of the samples at 600 nm was read with a spectrophotometric plate reader. The minimum inhibitory concentration (MIC) was defined as the lowest concentration of the antibiotic that inhibits bacterial growth.

### Genomic DNA isolation and whole genome re-sequencing (WGS)

Genomic DNA was isolated using Nucleospin Tissue Kit (Macherey Nagel). The resequencing library was constructed from the isolated genomic DNA using Kapa HyperPlus Kit (Roche) according to the manufacturer’s instruction. Then, the library was sequenced using a MiSeq Reagent Kit v3 (Illumina) in 600 cycle-pair-ended recipe on the MiSeq instrument (Illumina).

### Total RNA isolation and rRNA removal

Four milliliter of biologically duplicated cell cultures were harvested at the late exponential growth phase (OD_600 nm_ ~ 0.8) and treated with 8 mL of RNAprotect Bacteria Reagent (Qiagen) according to the manufacturer’s protocol. The pellets were stored at −80 °C. Total RNA was purified using an RNeasy MinElute Cleanup Kit (Qiagen) as follows. The pellet was resuspended with 500 μL of Buffer RLT containing 5 μL of β-mercaptoethanol. Resuspension was transferred to the screw-top micro-centrifuge tube containing a pre-measured amount (6 mm from the bottom of tube) of 1 mm diameter Zirconia/Silica beads (BioSpec Products). Then the cells were lysed by MagNA Lyser instrument (Roche) as followes: 30 sec at 6,000 × g, 1 min on ice, and 30 sec at 6,000 × g. The sample was transferred to a new tube without beads. 0.57 volumes of absolute ethanol to lysed cell was added and mixed by vortexing. Total RNA was purified using an RNeasy MinElute Cleanup Kit as described in the manufacturer’s instruction. DNA remained in total RNA was depleted by On-Column DNase I Digestion Set (Sigma) during purification. rRNA was subtracted from 1 μg of total RNA using Ribo-Zero rRNA Removal Kit for Gram-positive Bacteria (Illumina) according to the manufacturer’s instruction.

### Strand-specific RNA-seq (ssRNA-seq)

Strand-specific RNA-seq libraries were prepared from rRNA-depleted RNA sample using a Stranded RNA-seq Kit (Roche) according to the manufacturer’s instruction. Libraries were quantified by Qubit 2.0 fluorometer (Thermo) using Qubit dsDNA HS Assay Kit. Quality of libraries was analyzed by the BioAnalyzer 2100 with BioAnalyzer High Sensitivity DNA Kit (Agilent). The ssRNA-seq libraries were sequenced using a MiSeq Reagent Kit v3 (Illumina) in 150 cycle-pair-ended recipe on the MiSeq instrument (Illumina).

### Differential RNA-seq (dRNA-seq)

Differential RNA-seq library was prepared using the method described previously^30^. The rRNA-subtracted RNA was split into two samples. One of the samples was treated with 20 U of 5′ RNA polyphosphatase (Epicentre) at 37 °C for 60 min, while nuclease-free water was treated to the other sample. Dephosphorylated RNA adaptor was ligated to both polyphosphatase-treated and water-treated RNA samples with 1:3 molar ratio of RNA to adaptor by incubating at 37 °C for 90 min with 5 U of T4 RNA ligase (Epicentre). The RNA adaptor was dephosphorylated by 5 U of Antarctic Phosphatase (NEB) at 37 °C for 30 min followed by 70 °C for 15 min to remove any phosphate group at the 5′ end before use. The adaptor-ligated RNA samples were purified using 2.4-fold excess AMPure XP beads (Agencourt). cDNA was then synthesized from the purified RNA sample using SuperScriptase III RT (Invitrogen) with 3.125 pmol of random nonamer and excess oligo DNA was removed with 0.8 volumes of AMPure XP beads. cDNA was amplified by PCR reaction with Phusion High-Fidelity DNA Polymerase, P5 primer and P7 index primer. The amplification was stopped before reaching the plateau, typically at 19^th^ cycle. PCR products were purified with 0.8 volumes of AMPure XP beads and analyzed using Qubit fluorometer and BioAnalyzer. The dRNA-seq libraries were sequenced using a MiSeq Reagent Kit v3 (Illumina) in 150 cycle-pair-ended recipe on the MiSeq instrument (Illumina).

### Quantitative reverse transcription PCR (qRT-PCR)

Quantitative reverse transcription was performed from 4 ng of total RNA in 20 μL reaction using GoTaq 1-Step RT-qPCR system (Promega) according to the manufacturer’s instructions. 25 pmol of forward and reverse primers were included in each reaction and amplification was monitored in CFX Connect Real-Time PCR Detection System (BioRad) with following conditions: 37 °C for 15 min, 95 °C for 10 min followed by 40 cycles of 95 °C for 10 sec, 58 °C for 30 sec, and 72 °C for 30 sec.

### Rapid amplification of 5′ cDNA ends (5′ RACE)

One microgram of rRNA-depleted RNA was ligated with 50 pmol of Blocking RNA Adaptor using 10 U of T4 RNA ligase (Thermo) for 90 min at 37 °C followed by 10 min at 70 °C. Excess adaptors were removed by two volumes of AMPureXP beads as manufacturer’s instruction. Purified RNA sample was divided into two reactions for TAP treatment (+TAP) and negative control (−TAP). Two samples were incubated with or without TAP enzyme for 60 min at 37 °C. Two samples were then purified by ethanol precipitation. Purified RNA samples were ligated to 10 pmol of Amplification Adaptor using 10 U of T4 RNA ligase at 37 °C for 90 min and then 70 °C for 10 min. Excess adaptors were removed by AMPureXP beads and each sample was recovered in 10 μL of nuclease-free water. Eight microliters of the adaptor-ligated RNA was reverse-transcribed by SuperScript III RT (Invitrogen) with 50 ng of random hexamer. The synthesized cDNA was purified by AMPureXP beads followed by ethanol precipitation. Target genes were amplified by two sequential PCR amplifications. First, target was amplified from 2 μL of cDNA with 25 pmol of Amplification Primer and 10 pmol of target-specific reverse primer in 20 μL of PCR reaction. PCR products were then 100-fold diluted and re-amplified with each 10 pmol of Amplification Primer and target-specific second reverse primer. Amplified DNA samples were analyzed on 2% agarose gel and eluted from the gel for Sanger sequencing.

### Data processing

Sequencing data processing was done in CLC Genomics Workbench (CLC Bio). Raw reads were trimmed by Trim Sequence Tool in NGS Core Tools with quality limit of 0.05. Reads with more than two ambiguous nucleotides were discarded and the quality trimmed reads were mapped on reference genome sequence (NC_010079, NC_010063, and NC_012417) with following parameters; mismatch cost: 2, indel cost: 3, length and similarity fractions: 0.9. Reads mapped to the multiple genomic positions were mapped randomly for WGS and discarded for dRNA and ssRNA-seq. Variants were detected by Quality-based Variant Detection Tool using following parameters; neighborhood radius: 5, maximum gap and mismatch counts: 5, minimum neighborhood and central qualities: 30, minimum coverage: 10, minimum variant frequency: 10%, and maximum expected alleles: 4. Non-specific matches were ignored and bacterial genetic code was used. Variants in repeat region (i.e. rRNAs and transposases) were discarded. *De novo* genome assembly was done by de novo assembly tool in CLC Genomics Workbench with mismatch cost, insertion cost, deletion cost, similarity fraction, length fraction, and minimum contig length of 2, 3, 3,0.9, 0.9, and 1000, respectively (all other parameters were set as default or auto-detect mode). Expression levels were normalized by DESeq2 software package in Bioconductor version 3.4 on R workspace from the mapped read counts of genes calculated from Transcriptome Analysis Tool in CLC Genomics Workbench. TSS determination was done with in-house python scripts. Motif search was done in interactive MEME suite version 4.11.1 and the motifs were directly submitted to Tomtom tool in MEME suite for comparison of the known bacterial transcription factor binding motifs^77^.

### Determination of TSS

Mapping file of dRNA-seq was exported into bam file format and converted into profile (gff file format). The mapping profile was analyzed by in-house analysis pipeline on python 2.0 workspace (Supplementary Fig. S14). The −TAP and +TAP profiles were normalized nucleotide-by-nucleotide using following equation (Eq. 1).

1 $${normalized}\,{read}\,{coun}{{t}}_{{position}}={read}\,{coun}{{t}}_{{position}}\times \frac{{10}^{9}}{{total}\,{mapped}\,{bases}}$$

Then, fold-enrichment values were calculated by dividing +TAP count by −TAP count throughout the genome. If enrichment was higher than fold-change cutoff or there was +TAP signal but not in −TAP, the genomic positions were selected as a possible TSS. The fold-change cutoff was adjusted typical value within 1.5 and 2 by manually inspecting output TSSs to minimize manual curation step. Among genomic position selected, positions with + TAP signal lower than noise cutoff (50th percentile of signals from all genomic positions) were discarded. Then adjacent TSS positions were grouped into long TSS regions. TSS regions shorter than length cutoff (10 nt) were discarded. The logic of length cutoff is that short TSS regions are likely to be false positives, because sequencing reads had a specific length. In some cases, TSS regions were divided into several pieces because of the shape of −TAP profile (Supplementary Fig. S14; merge false-split). Those regions were rejoined together. In bacterial transcription, TSS can move few nucleotides in a single promoter. Thus, 5′ end of TSS region was curated to a position where +TAP profile of 1 nt upstream genomic position is no higher than 20% of the position. The 5′ end was curated within 5 nucleotides. To find alternative TSSs located in one TSS region, an increase (2-fold) of +TAP signal at least 5 nucleotides in a TSS region was detected. Alternative TSSs and 5′ end of TSS regions were exported to a gff file format. To remove any errors in computational assignment, all the TSSs determined were curated by manual inspection. The TSSs determined in four different antibiotic treatments were compared to obtain full set of TSSs in the strain. TSSs from different conditions with very close positions were regarded as the same TSSs using 10 nt sliding window. Total TSSs were categorized into 5 types and assigned to downstream gene if there exists a gene within 300 nt. In parallel, TSSs from individual treatment were also assigned to trace conditional origin of TSSs. Then, alternative transcription was detected and listed from primary and secondary TSS. TSS previously detected in *S. aureus* MW2^31^ were mapped on TCH1516 genome and compared with present TSSs using 3 nt window.

### Promoter analysis

DNA sequence from 50 nt upstream to 10 nt downstream of the determined TSSs were extracted and analyzed by MEME version 4.11.1 to find -10 and -35 element (*p*-value cutoff = 0.05, MEME).

### Hierarchical Clustering

Genes were clustered according to their expression patterns using hierarchical clustering method. Log_2_Fold-change values were used. After clustering, genes related with euclidean distance less than 2.5 were determined as a gene cluster.

### Pathway Enrichment Analysis

Pathway enrichment analysis was done by ClueGO plug-in version 2.2.4 of Cytoscape version 3.3.0. DEGs were used as an input (up and down-regulated DEGs were analyzed separately). GO biological function and KEGG database were used for analysis with following parameters (GO-term fusion was used and terms with less than 4 genes or less than 1% of composing genes were ignored; Kappa (connectivity) score, 0.45; Network style, “significance”).

### Data availability

WGS and RNA sequencing data were deposited in the European Nucleotide Archive (accession number PRJEB23980).

## Electronic supplementary material

Dataset S1

Dataset S2

Dataset S3

Dataset S4

Dataset S5

Dataset S6

Dataset S7

Dataset S8

Dataset S9

Supplementary Information
