# Supplementary material for: Genome-scale analysis of Methicillin-resistant Staphylococcus aureus USA300 reveals a tradeoff between pathogenesis and drug resistance
Source: Sci Rep. 2018 Feb 2;8:2215. doi: 10.1038/s41598-018-20661-1 (PMC5797083; doi:10.1038/s41598-018-20661-1)
Supplement: Supplementary file 10 — Supplementary Information [file 41598_2018_20661_MOESM10_ESM.pdf]

SUPPLEMENTARY INFORMATION

Genome-scale analysis of Methicillin-resistant *Staphylococcus aureus* USA300 reveals a tradeoff between pathogenesis and drug resistance

Donghui Choe[1], Richard Szubin[2], Samira Dahesh[3], Suhyung Cho[1,4], Victor Nizet[3]*, Bernhard Palsson[2, 3]*, and Byung-Kwan Cho[1, 4]*

[1]Department of Biological Sciences, Korea Advanced Institute of Science and Technology, Daejeon 34141, Republic of Korea

[2]Department of Bioengineering, University of California San Diego, La Jolla, CA92122

[3]University of California San Diego School of Medicine, La Jolla, CA92122

[4]KI for the BioCentury, Korea Advanced Institute of Science and Technology, Daejeon 34141, Republic of Korea

*Correspondence should be addressed to B.-K.C. (bcho@kaist.ac.kr), B.O.P. (bpalsson@ucsd.edu), and V.N. (vnizet@ucsd.edu)

LIST OF CONTENTS

Supplementary Figures S1 to S14

Extended references

SUPPLEMENTARY FIGURES

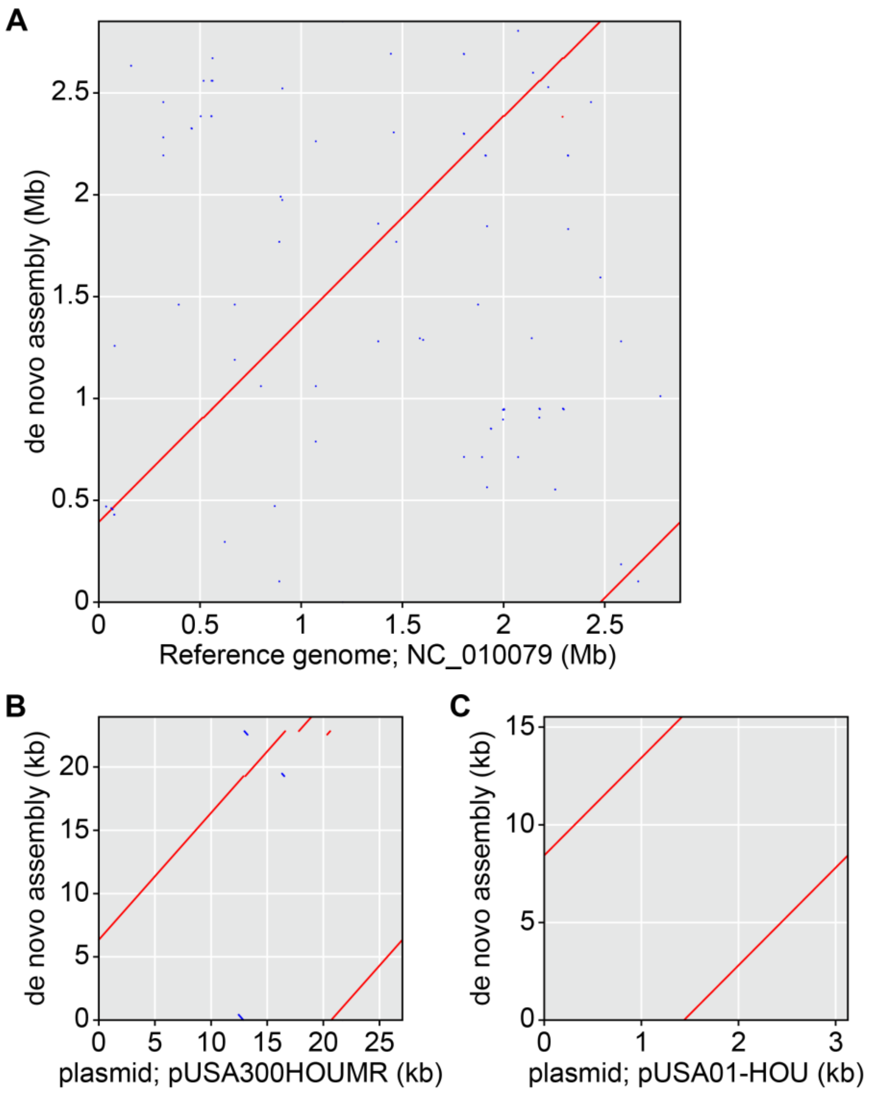

Fig. S1. Alignment of de novo assembled contigs and reference genome. All 29 contigs were aligned to reference genome without macroscale genome rearrangement (such as inversion).

A

Antibiotic	MIC (µg/mL)
Linezolid	0.5
Nafcillin	2.5
Vancomycin	0.6

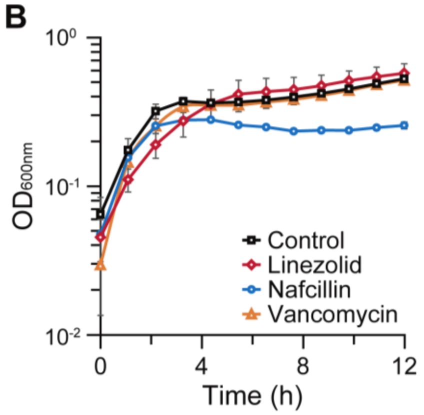

Fig. S2. Antibiotics sensitivity of *S. aureus* USA300_TCH1516. **(A)** Minimum Inhibitory concentrations (MIC) of linezolid, nafcillin, and vancomycin were measured. **(B)** The cells were grown under the given antibiotic treatments with sub-inhibitory concentrations. Antibiotic treatments did not affect the strain’s growth.

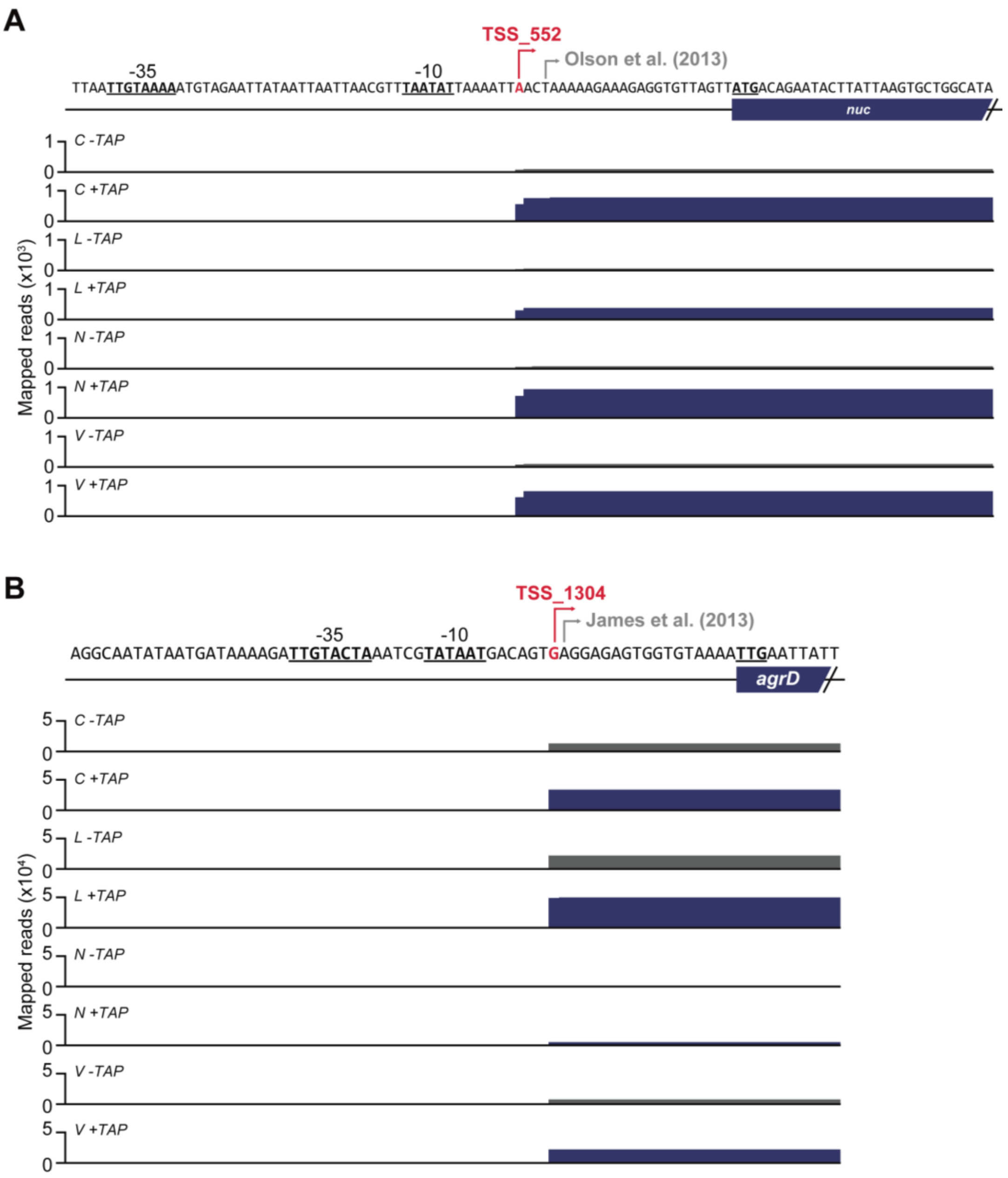

Fig. S3. Comparison of TSSs determined in this study with previously reported TSSs. (A) TSS_552 was coinsided with the previously determined TSS (1). **(B)** Well-studied *agr* P2 promoter (2) and TSS_1304 agree with 1 nt difference.

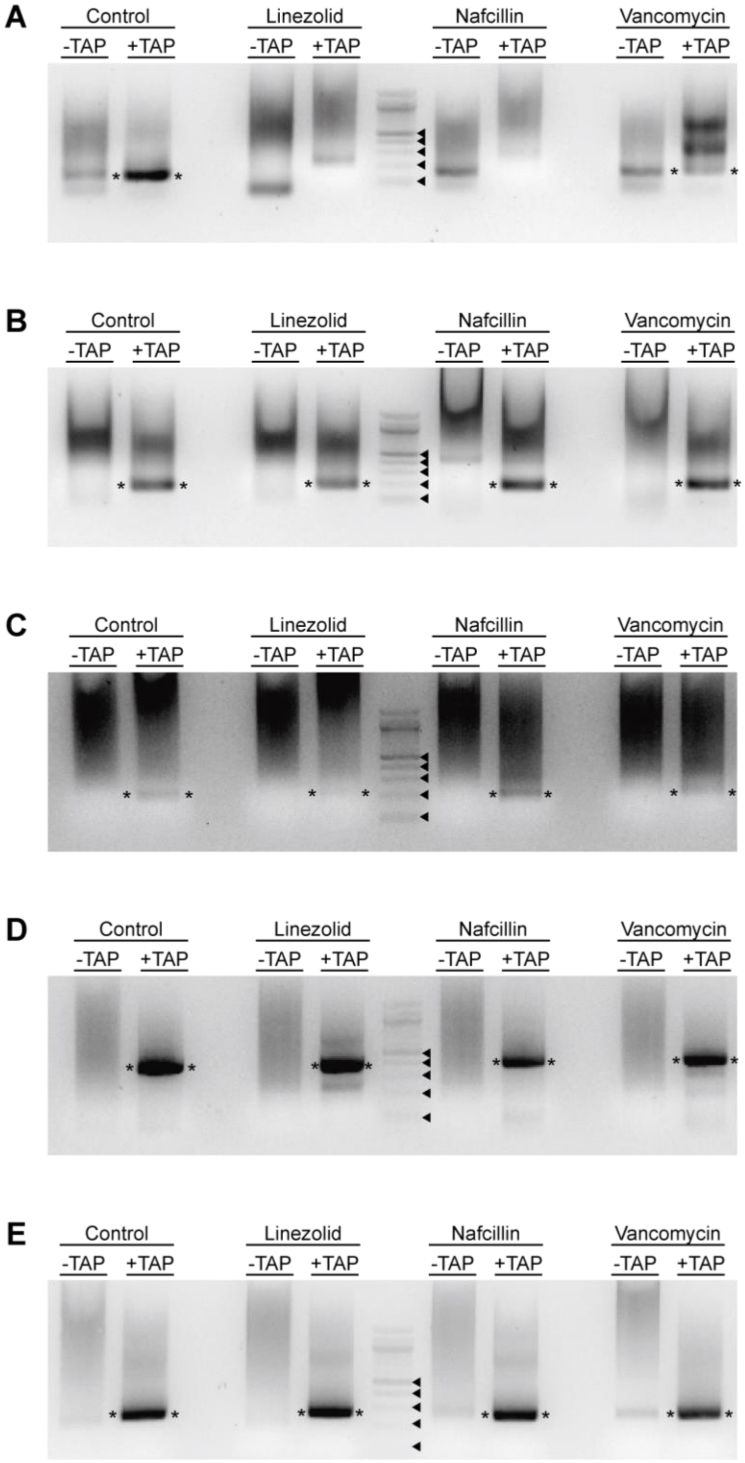
A
Control
-TAP
+TAP
Linezolid
-TAP
+TAP
Nafcillin
-TAP
+TAP
Vancomycin
-TAP
+TAP
B
Control
-TAP
+TAP
Linezolid
-TAP
+TAP
Nafcillin
-TAP
+TAP
Vancomycin
-TAP
+TAP
C
Control
-TAP
+TAP
Linezolid
-TAP
+TAP
Nafcillin
-TAP
+TAP
Vancomycin
-TAP
+TAP
D
Control
-TAP
+TAP
Linezolid
-TAP
+TAP
Nafcillin
-TAP
+TAP
Vancomycin
-TAP
+TAP
E
Control
-TAP
+TAP
Linezolid
-TAP
+TAP
Nafcillin
-TAP
+TAP
Vancomycin
-TAP
+TAP

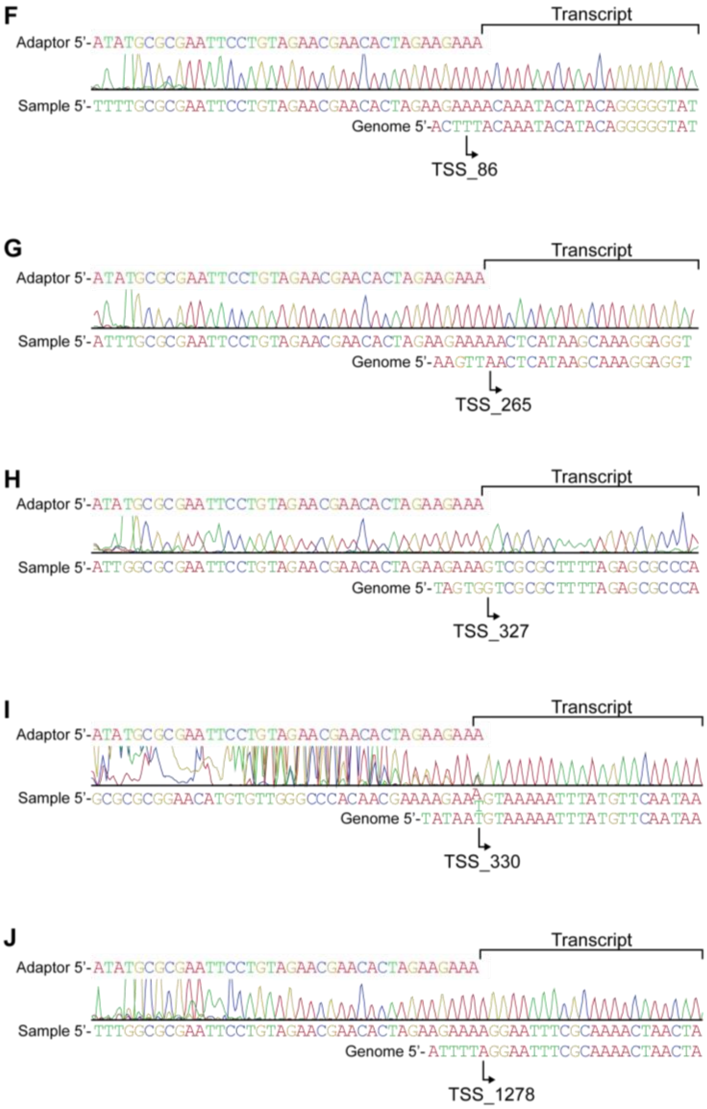

Fig. S4. 5'-RACE confirmation of TSSs. TSS determined by dRNA-seq was confirmed by 5'-RACE. **(A)** TSS_86 (Spa), **(B)** TSS_265 (PSMα), **(C)** TSS_327 (RplK), **(D)** TSS_330 (RpoB), and **(E)** TSS_1278 (Chp) were analyzed on agarose gel. Asterisk (*) indicates the predicted size of amplicon. Bands of 100 bp DNA ladder are marked as (▲); 100, 200, 300, 400, and 500 bp (from the bottom to top). The amplicons were isolated and further analyzed by Sanger sequencing. Sequencing results of **(F)** TSS_86, **(G)** TSS_265, **(H)** TSS_327, **(I)** TSS_330, and **(J)** TSS_1278 were aligned to genomic sequence and position of TSS.

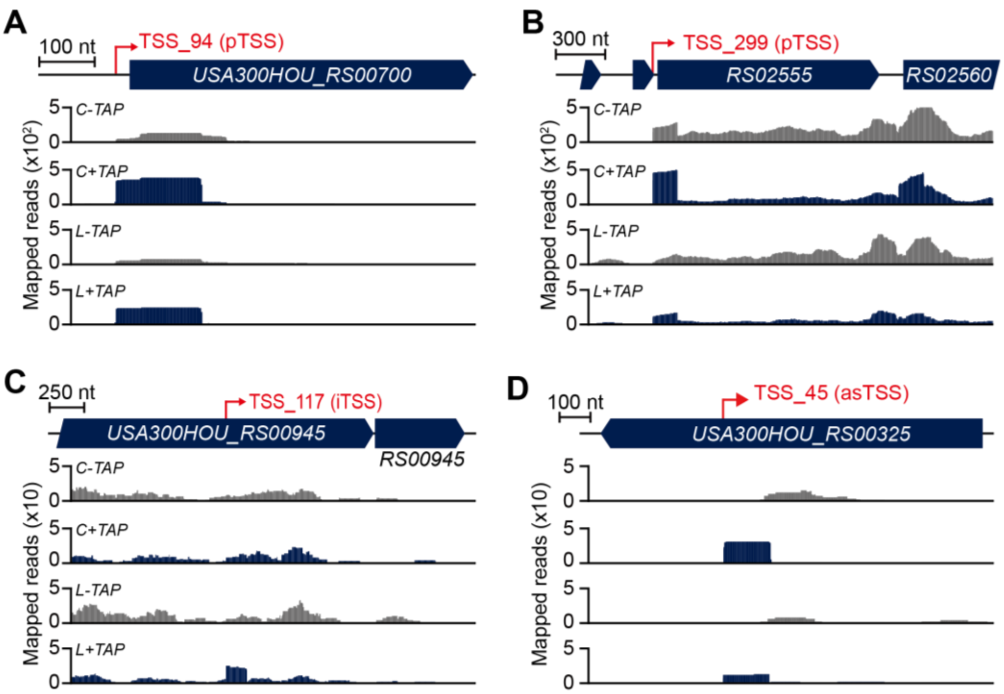

Fig. S5. Examples of dRNA-seq profiles for the TSS categories. Profiles show examples of **(A)** pTSS in an intergenic region, **(B)** pTSS in a gene, **(C)** iTSS, and **(D)** as TSS. Only control and linezolid conditions were shown.

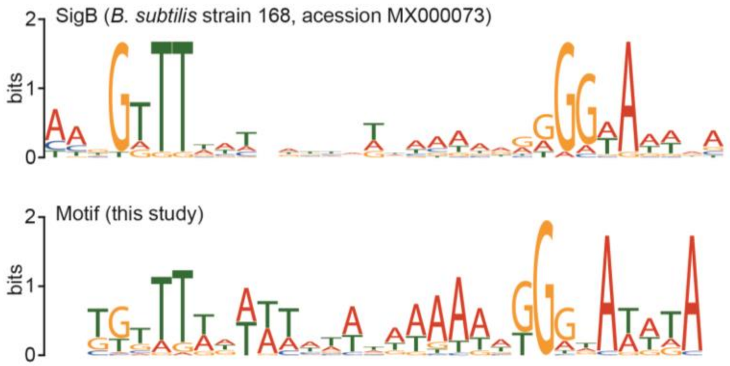

Fig. S6. Comparison between SigB-binding motifs of *Bacillus subtilis* and found from 26 TSSs. Upper panel shows alignment of SigB binding motifs in *Bacillus subtilis* strain 168 and lower panel shows alignment of motifs found upstream of 26 TSS.

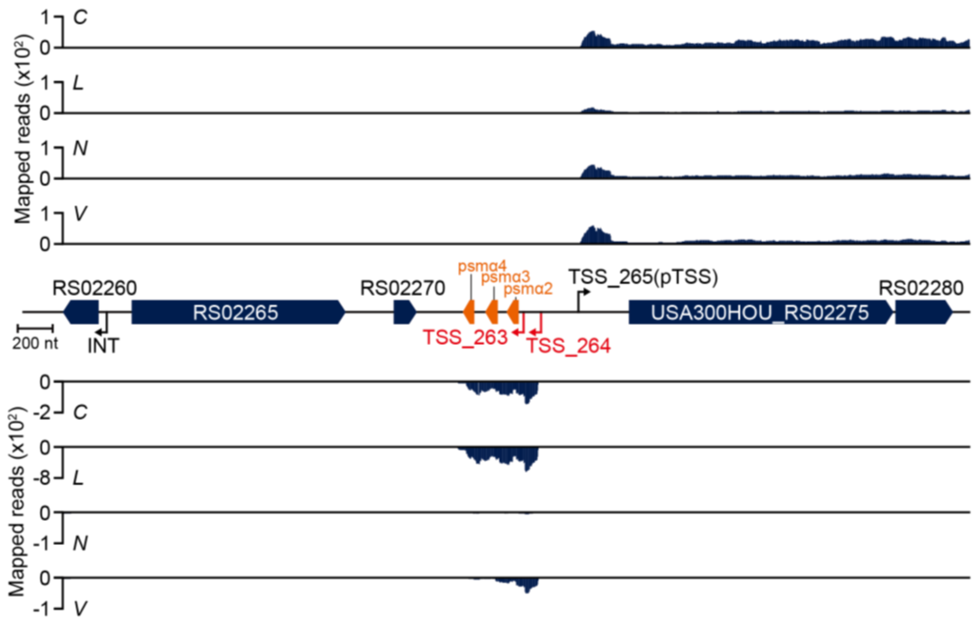

Fig. S7. ssRNA-seq profile of PSMα2, 3, and 4. The profile shows transcription of novel transcript PSMα (orange arrows) by TSS_263 and TSS_264.

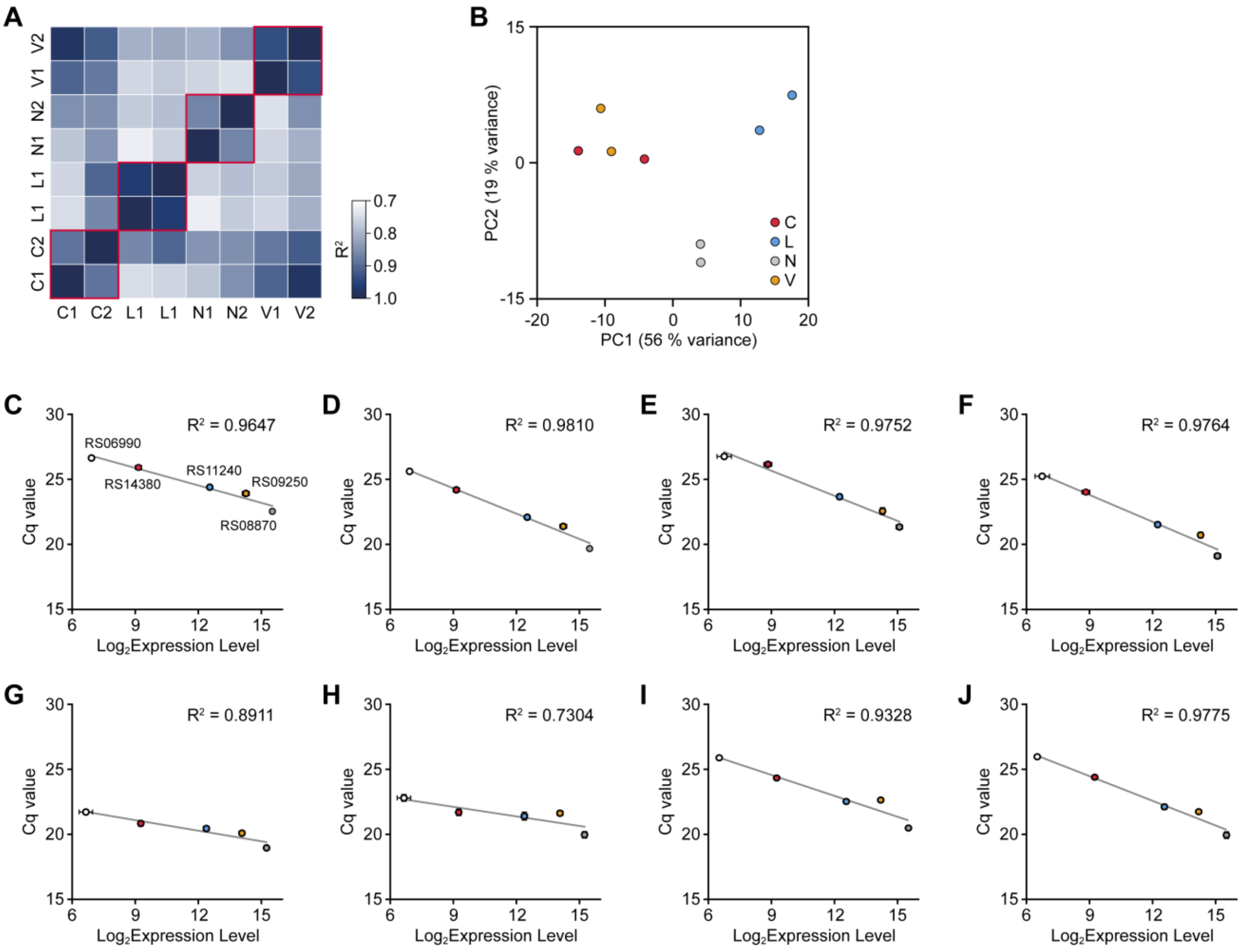

Fig. S8. Reproducibility, principal component analysis, and qRT-PCR confirmation of ssRNA-seq. (A) Pearson correlation (R^2) between each replicates and conditions of RNA-seq. **(B)** Principal component analysis (PCA) showing geometric distance between samples. Two replicates correlated with correlation constant over 0.86. In PCA, samples from the same condition locate closely to each other, except one replicates of control and vancomycin condition. **(C-J)**, Correlation between expression levels determined by ssRNA-seq and qRT-PCR; Log_2(expression level, ssRNA-seq) versus Cq value determined by qRT-PCR of **(C)** control replicate 1, **(D)** control replicate 2, **(E)** linezolid replicate 1, **(F)** linezolid replicate 2, **(G)** nafcillin replicate 1, **(H)** nafcillin replicate 2, **(I)** vancomycin replicate 1, and **(J)** vancomycin replicate 2.

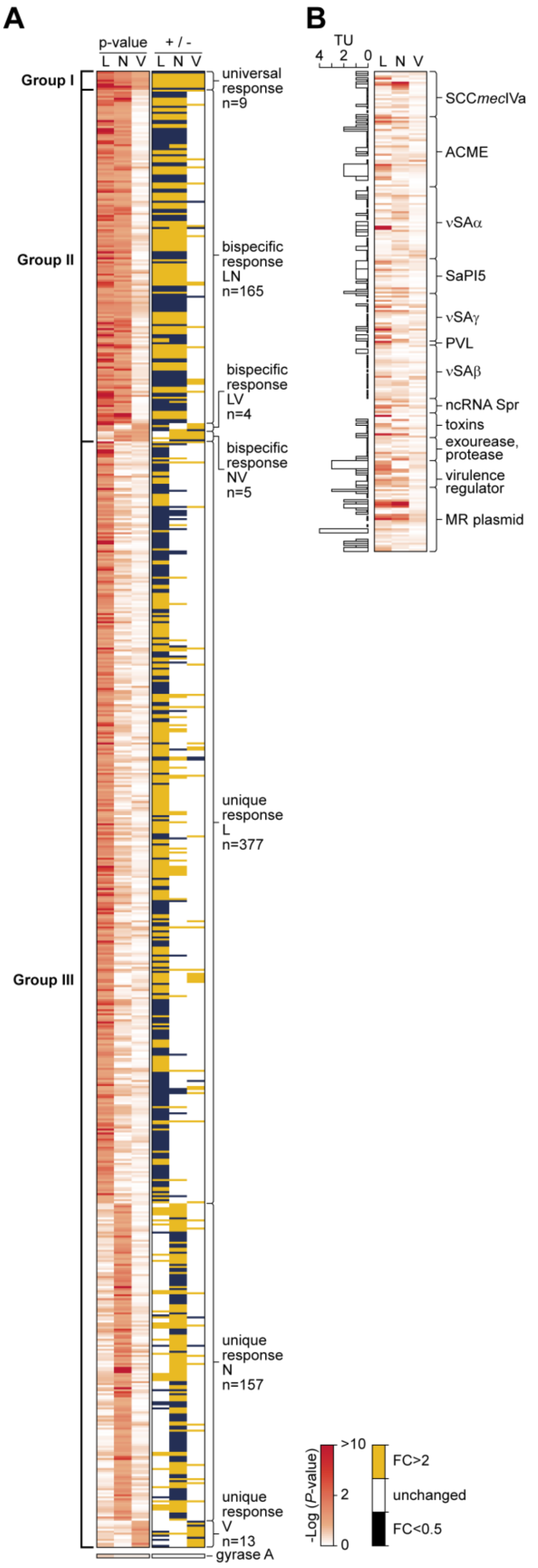

Fig. S9. Differential expression of genes in response to antibiotics treatments. (A) 730 genes considered as DEGs with statistical significance lower than 0.01 and expression change greater than two-fold in response to antibiotics. DEGs were divided into three large groups (Group I, II, and III). **(B)** Total 233 genes in 134 operons responsible for virulence and antibiotics resistance. Genes in mobile genetic elements (MGE) and MR plasmid are included. In addition, virulence regulators and ncRNAs found in dRNA-seq are shown together.

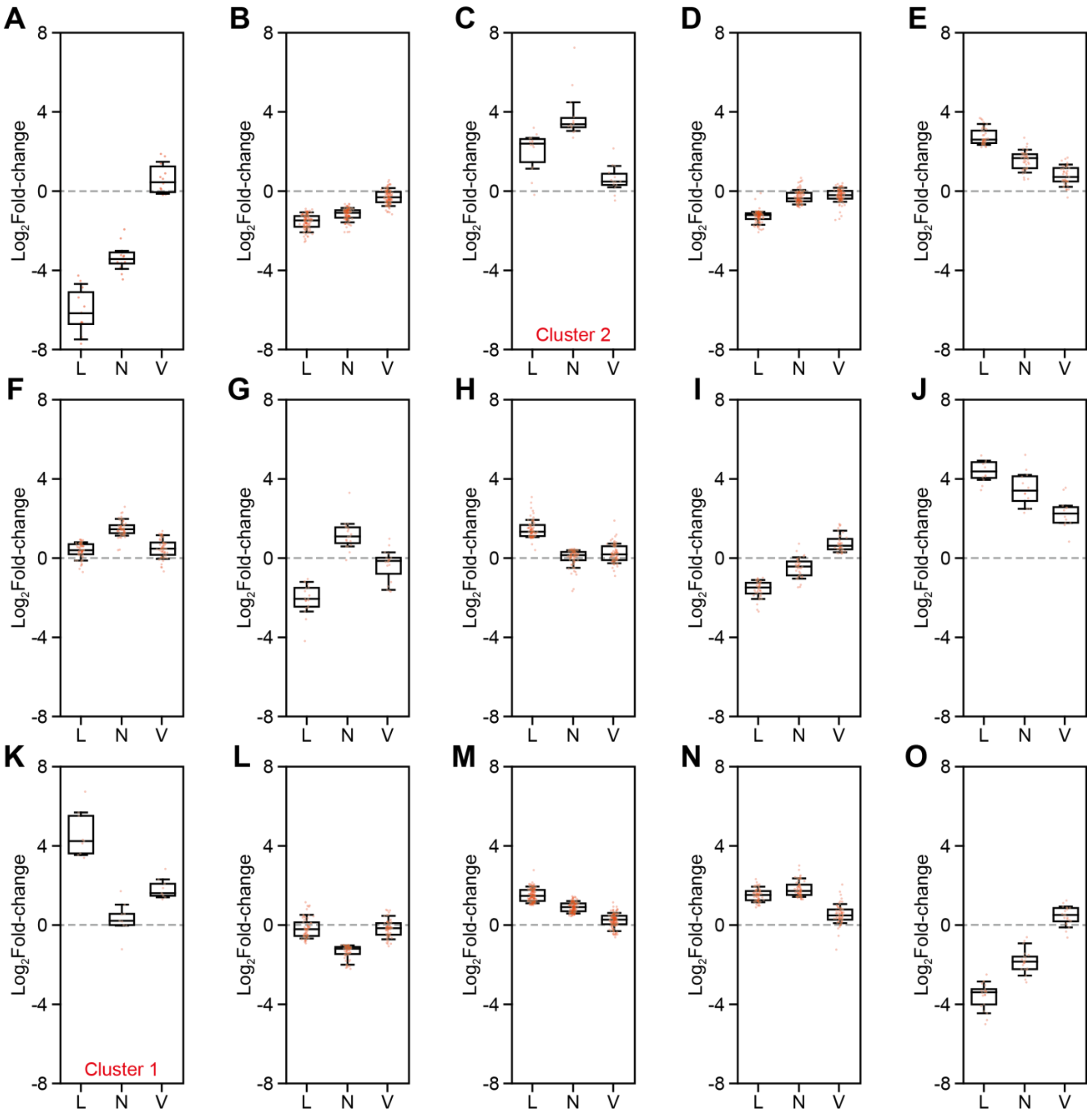

Fig. S10. Distribution of expression change of genes in clusters determined.

Expression changes of ten clusters determined by hierarchical clustering are shown.

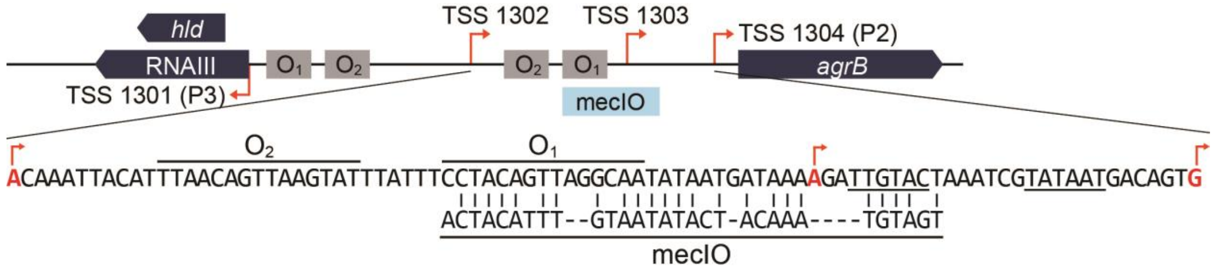

Fig. S11. Transcription start and regulatory elements found in *agr* locus. Two pairs of agr binding operators (O1 and O2) are located upstream of P2 and P3. Two alternative transcriptions of *agrBDCA* operon were observed. One of the alternative transcription (TSS_1302) may bypass the regulation of agr-regulation by locating upstream of agr operators. Agr operator 2 had 65% similarity with methicillin response regulator (MecI) binding site.

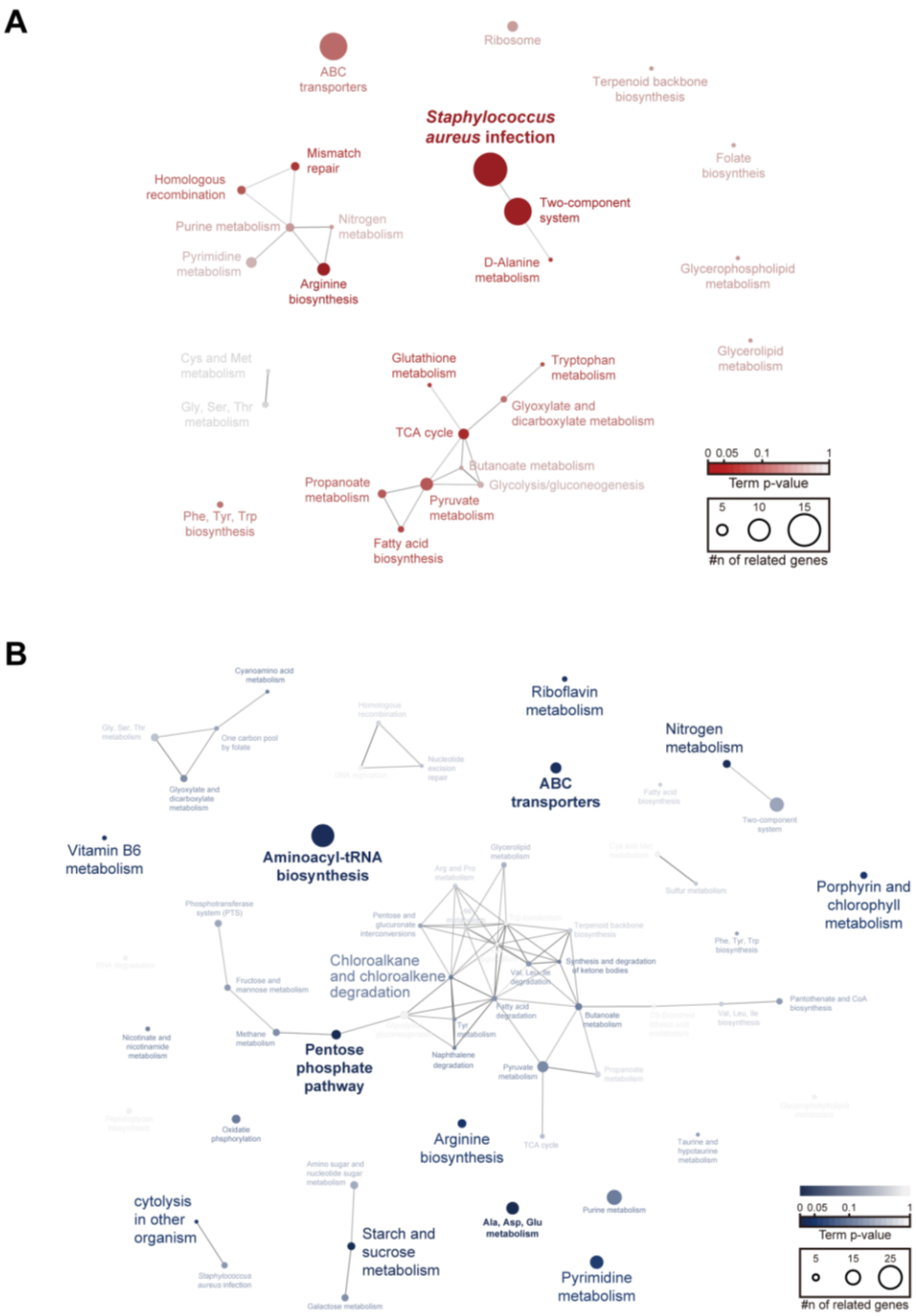

Fig. S12. Network of enriched KEGG pathway in linezolid treatment. Networks show the enriched pathways of **(A)** up regulated genes and **(B)** down regulated genes. Size of nodes was determined by number of genes found in the pathway. Color of nodes was determined by *p*-value of each node. Length of edge connecting to nodes had no informational content, except connection.

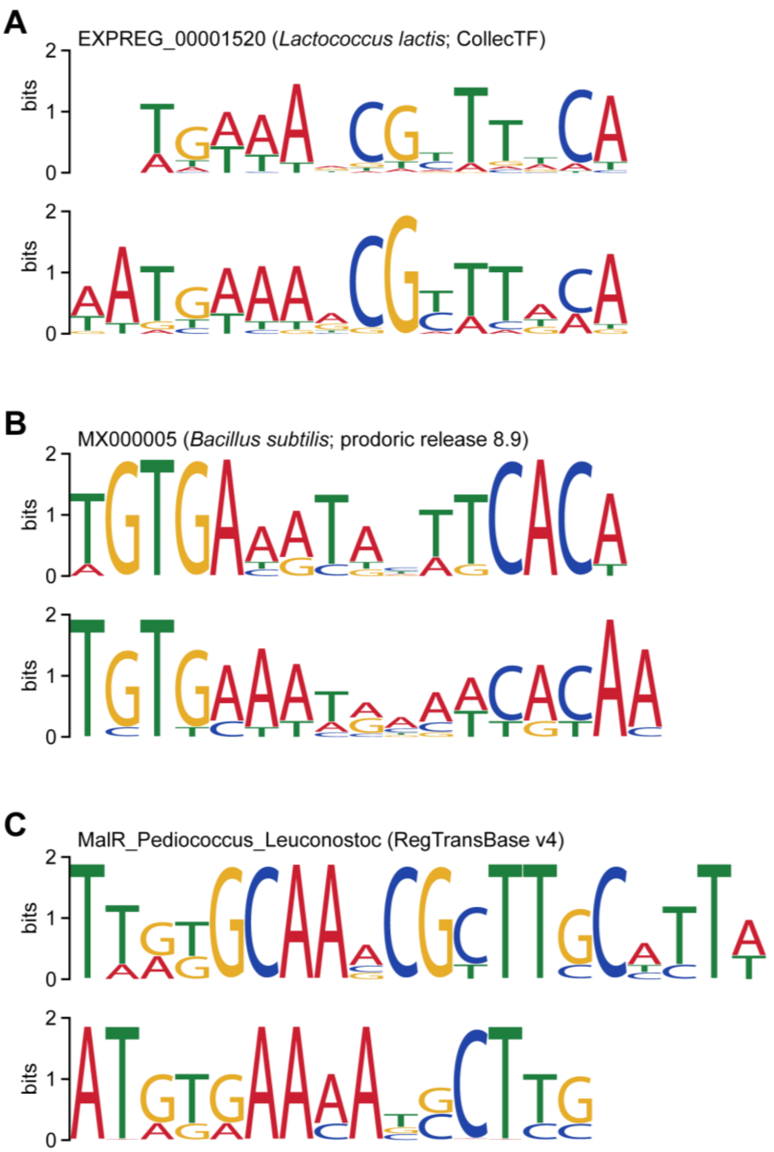

Fig. S13. Motif search of the up or down regulated genes grouped by KEGG pathways. Comparison of **(A)** CcpA, **(B)** Fnr, and **(C)** MalR binding motifs of database (upper) with found in this study (lower).

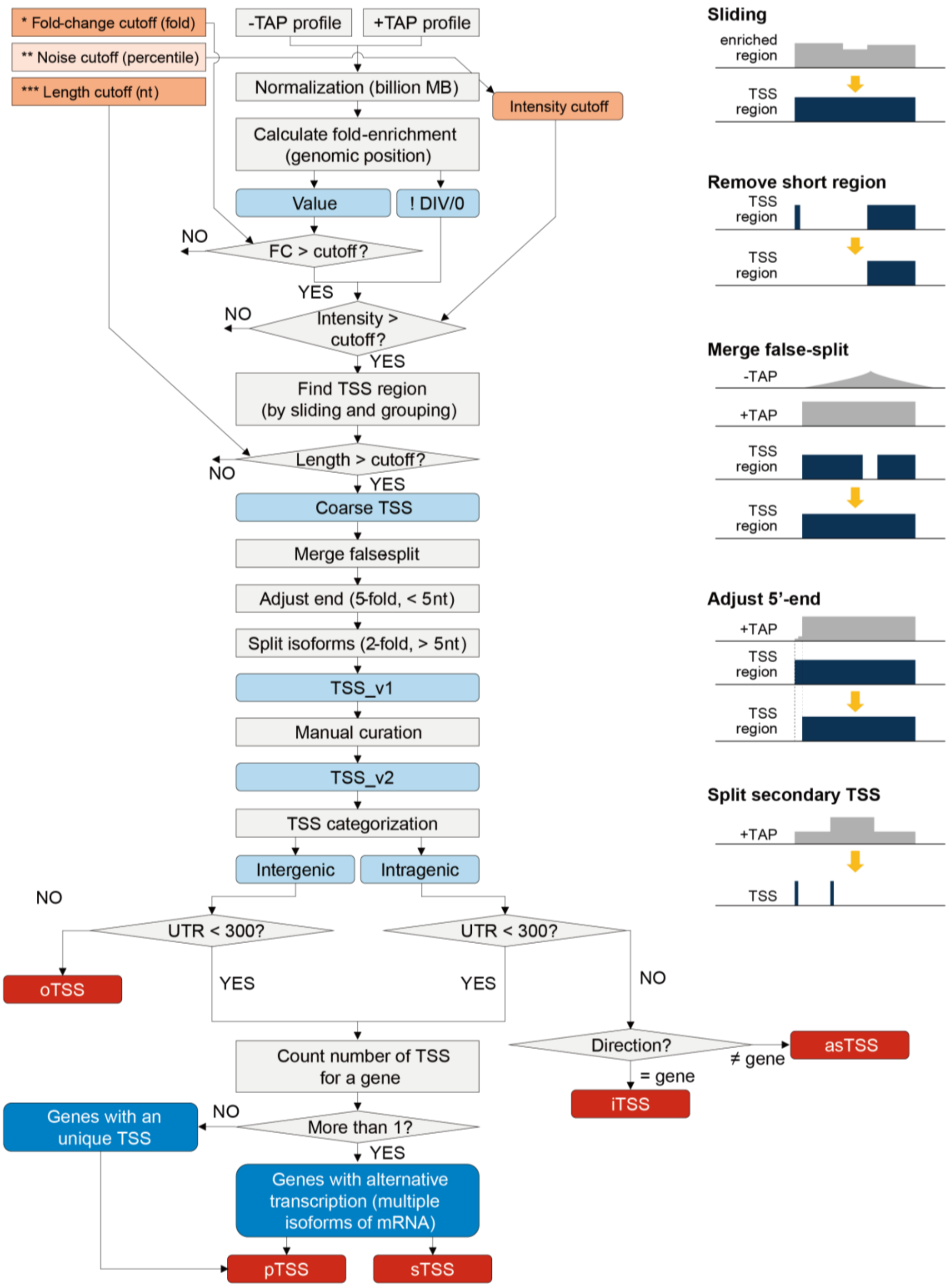

Fig. S14. Determination and categorization of TSSs in *S. aureus* USA300 TCH1516.

Schematic description of in-house analysis pipeline.

EXTENDED REFERENCES

1. Olson, M.E., Nygaard, T.K., Ackermann, L., Watkins, R.L., Zurek, O.W., Pallister, K.B., Griffith, S., Kiedrowski, M.R., Flack, C.E., Kavanaugh, J.S. *et al.* (2013) Staphylococcus aureus nuclease is an SaeRS-dependent virulence factor. *Infect Immun*, **81**, 1316-1324.
2. James, E.H., Edwards, A.M. and Wigneshweraraj, S. (2013) Transcriptional downregulation of agr expression in *Staphylococcus aureus* during growth in human serum can be overcome by constitutively active mutant forms of the sensor kinase AgrC. *FEMS Microbiol Lett*, **349**, 153-162.
